# The genes regulating sensitivity of tumor cells to T cell-mediated killing: could they be potential personalized immunotherapeutic targets in head and neck squamous cell carcinoma?

**DOI:** 10.1007/s12672-023-00806-z

**Published:** 2023-11-05

**Authors:** Shaonan Hu, Heng Duan, Yongtao Lu, Shaohong Huang

**Affiliations:** Stomatological Hospital, School of Stomatology, Southern Medical University, 366 Jiangnan South Avenue, Haizhu District, Guangzhou, 510280 Guangdong China

**Keywords:** STTK genes, HNSC, Tumor immunity, Personalized medicine, Informatics

## Abstract

**Objective:**

To identify the pivotal genes, specifically the STTK genes, that govern the sensitivity of tumor cells to T cell-mediated killing in Head and Neck Squamous Cell Carcinoma (HNSC).

**Methods:**

The differentially expressed genes (DEGs) in HNSC and STTK genes were overlapped to obtain the DE-STTK genes. Univariate and LASSO regression analyses were conducted to identify the pivotal DE-STTK genes that serve as hubs in HNSC (i.e., hub DE-STTK genes). The risk model was established to divide HNSC tumor samples into high- and low-risk groups based on the hub DE-STTK genes. Further investigations were carried out by examing the expression level, prognostic values, diagnostic values, enriched signaling pathways, correlation with tumor mutation burden (TMB), and association with tumor immune infiltration cells (TIICs).

**Results:**

A total of 71 genes were found to be overlapped between DEGs in HNSC and STTK genes. Lasso regression analysis identified 9 hub genes which were MYF6, AATF, AURKA, CXCL9, DPM2, MYO1B, NCBP2, TNFRSF12A, and TRAF1. The network analysis of hub DE-STTK genes-pathway reveals that these 9 hub genes exhibit enrichment in multiple signaling pathways, including toll-like receptor signaling, TNF signaling, NF-kappa B signaling, cytokine-cytokine receptor interaction, spliceosome, mRNA surveillance pathway, nucleocytoplasmic transport, GPI-anchor biosynthesis, as well as N-Glycan biosynthesis. The Pearson correlation analysis showed that the majority of correlations between 9 hub DE-STTK genes and immune cells were positive.

**Conclusion:**

The 9 identified hub DE-STTK genes (MYF6, AATF, AURKA, CXCL9, DPM2, MYO1B, NCBP2, TNFRSF12A, and TRAF1) are presumptively implicated in the modulation of tumor immunity in HNSC. These genes, along with their enriched pathways, hold promise as potential personalized immunotherapeutic targets for the treatment of HNSC, thereby offering novel avenues for therapeutic intervention in this malignancy.

## Introduction

HNSC, a form of cancer affecting the head and neck region, is a major public health concern., ranking as the sixth prominent contributing factor of cancer-related mortality worldwide. Typically, the first line of treatment for HNSC involves surgery to remove the tumor. This is often followed by additional treatments such as radiation therapy or a combination of chemotherapy and radiation therapy (chemoradiotherapy) to target any remaining cancer cells and reduce the risk of recurrence [1]. Cetuximab, which is an antibody that targets the epidermal growth factor receptor (EGFR), initially obtained FDA approval for its combination with radiotherapy based on a randomized controlled phase III clinical trial conducted by Bonner et al. in 2006. The study revealed that the combination of cetuximab with radiotherapy improved the survival rate and locoregional control in patients with locoregional advanced HNSC, resulting in the approval of this combination therapy by FDA for locally/regionally advanced HNSC [2]. Subsequent randomized trials conducted outside the United States revealed that the addition of cetuximab to cisplatin and 5-fluorouracil chemotherapy increased the OS of patients with recurrent locoregional or metastatic HNSC. The combination therapy also significantly enhanced the response rates (35.6% versus 19.5%) compared to chemotherapy alone. In light of these discoveries, the FDA granted additional approval in 2011 for the use of cetuximab alongside platinum-based chemotherapy as the initial treatment option for patients with recurrent locoregional or metastatic HNSC [3]. This approval provides an important new treatment option for HNSC. Despite the presence of numerous clinical treatment modalities and the continuous improvement in therapeutic efficacy [4, 5], the 5-year overall survival rate for patients with HNSC has yet to surpass 60%. Furthermore, the treatment strategies have demonstrated inherent limitations (such as the emergence of multiple complications and the development of treatment resistance) [6], indicating that the management of HNSC still encounters intricate challenges.

Immunotherapy has been extensively utilized in the management of solid tumors in recent years. Immune checkpoints constitute a complex network of immunosuppressive pathways inherent in the immune system, supervising the maintenance of self-tolerance and the management of natural immune responses to ensure that immune system activation remains within normal boundaries. The overexpression of immune checkpoint molecules leading to the suppression of immune function represents a pivotal mechanism facilitating immune evasion in certain tumor types. Immune checkpoint inhibitors (ICIs), a series of immunomodulatory antibodies, have emerged as a promising therapeutic approach capable of enhancing the host immune response against tumors, thereby exhibiting considerable efficacy in the treatment of cancer and improving patients' prognoses [7]. Currently, the FDA has granted approval for two immune ICIs (i.e., pembrolizumab and nivolumab), as therapeutic treatment alternatives for individuals with cisplatin-refractory recurrent or metastatic HNSC.

ICIs have been widely used in the clinical management of HNSC in recent years [8–10]. Although blockade of ICIs has emerged as an effective immunotherapy for HNSC, with a subset of patients demonstrating significant clinical benefits, immune resistance and the occurrence of adverse effects pose challenges in the current landscape of immunotherapy for HNSC [11]. The most prevalent adverse events associated with immunotherapy in patients with HNSC encompass a range of immune-related manifestations, including dermatological manifestations such as rash, gastrointestinal disturbances like diarrhea, thyroid dysfunction, and pulmonary inflammation such as pneumonitis. Other commonly reported effects include infusion-related reactions, fatigue, and reduced appetite. Specifically, CTLA-4 inhibitors have a higher incidence of colitis and hypophysitis, while PD-1 inhibitors are more frequently implicated in thyroiditis and pneumonitis. In general, most adverse effects can be effectively managed by administering corticosteroids or other immunosuppressive agents over a period of several weeks to months. It is worth noting, however, that the effects on the endocrine system may be irreversible. Consequently, meticulous oversight and management of these adverse events are essential for optimizing patient outcomes [12, 13]. It is challenging to identify the eligible patient subgroups with a higher likelihood of benefiting from immunotherapy prior to treatment initiation. Even with identical immunotherapy regimen applied, the treatment effects vary among different patients, resulting in over-treatment or under-treatment in clinical practice [9]. The tumor cell killing mediated by T cells is a pivotal element of tumor immunotherapy. However, the tumor microenvironment induced T-cell exhaustion has been recognized to decisively contribute to immune evasion mechanisms observed in cancer patients [10]. Consequently, enhancing the sensitivity of tumor cells to T-cell mediated killing represents a vital approach in augmenting the immunotherapy effectiveness for patients with HNSC. Studies have substantiated the pivotal involvement of genes involved in modulating tumor cell sensitivity to T-cell mediated killing (STTK-genes), in the realm of tumor immunotherapy. Hence, the identification of such genes holds potential for predicting the immune landscape and prognostic profiles of patients with HNSC. Thus, stratifying patients appropriately is an urgent issue to be addressed, which can help to enhance precision patient management and individualized therapeutic interventions while optimizing the efficiency of medical resource allocation. The comprehensive molecular characteristics and immune profile assessment suggest that the integration of prognostic biomarkers in clinical management offers potential solutions to address challenges in immunotherapy and extend patient survival [1].

To the authors’ knowledge, rare reports aimed on identifying if STTK genes could be involved in the tumor immunity of HNSC. In this study, we utilized a series of computational biology analyses to identify hub genes regulating tumor cell sensitivity to T cell killing that are differentially expressed in HNSC. We examined the prognostic value of these genes, established a risk model using them, evaluated enriched pathways, and analyzed correlations with tumor infiltrating immune cells. Through integrated analyses, we aimed to gain novel insights into genes and pathways involved in modulating tumor immunity in HNSC.

## Materials and methods

### Study design of the current research

**Fig. 1:**
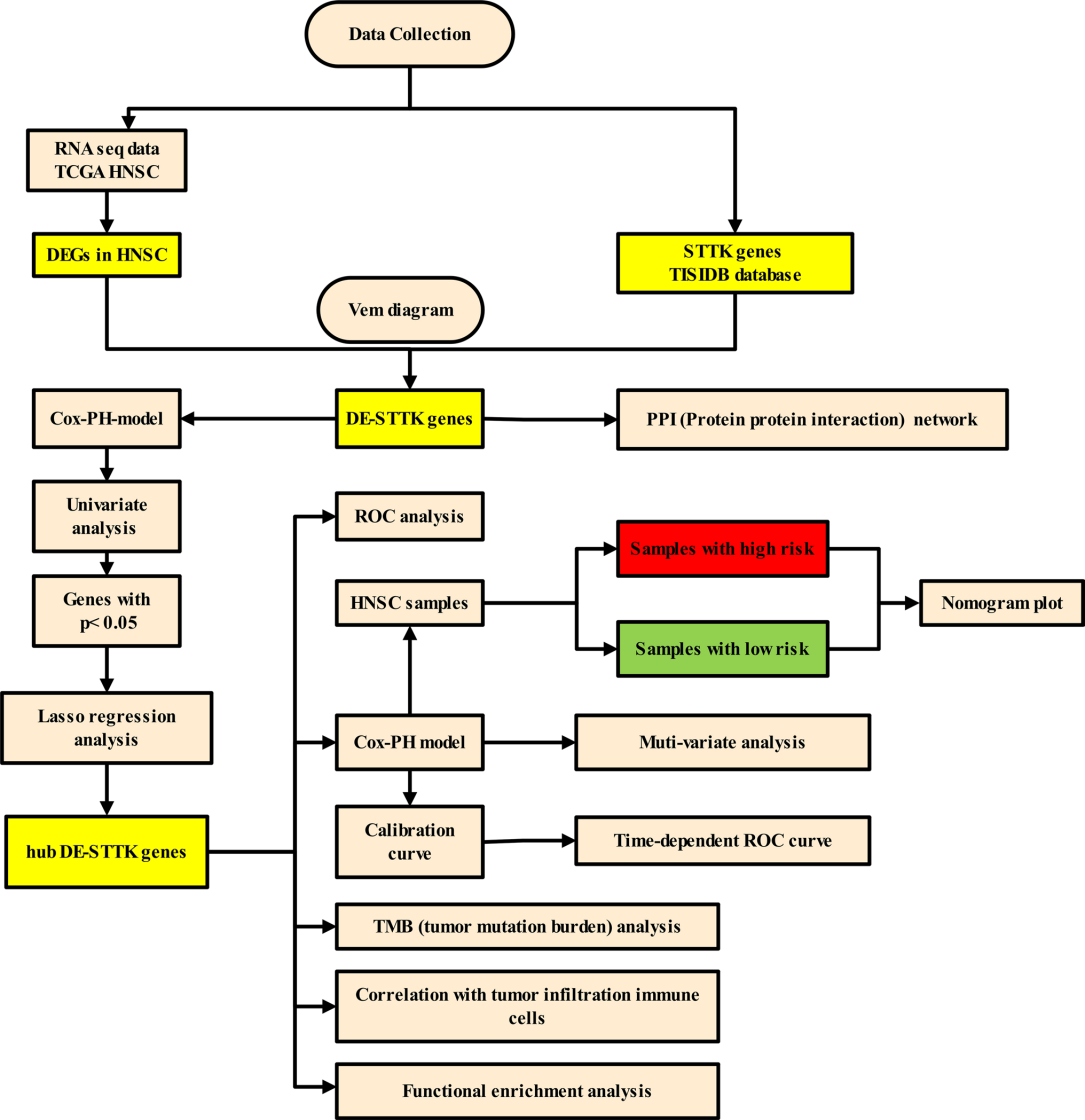
A computational workflow to identify candidate genes and pathways regulating tumor cell sensitivity to T cell killing in HNSC

In this study, a computational workflow was employed (shown in Fig. 1) to identify candidate genes and pathways that play a role in determining the tumor cell sensitivity to T cell killing in HNSC. RNA-sequencing data of HNSC samples were obtained from The Cancer Genome Atlas (TCGA) along with curated gene signatures of T cell-modulating genes from the TISIDB database. Multiple bioinformatics techniques including differential expression analysis, regression modeling, pathway analysis, prognostic modeling, and immune correlation analysis were applied in an integrative step-wise manner. This systematic computational approach enabled the identification of key differentially expressed T cell-sensitizing genes and associated signaling pathways that may participate in shaping anti-tumor immune responses in HNSC.

### Datasets procurement

The RNA-seq dataset of HTseq for Head and Neck Squamous Cell Carcinoma (HNSC) was downloaded from TCGA-GDC (https://portal.gdc.cancer.gov/), and then data sets with TPM (Transcripts Per Million) as the expression value type were integrated from all samples. Samples numbered 01 and 11 were screened, where the number 01 sample was set as the Tumor group (e.g., TCGA-CV-6942-01A, TCGA-CN-6024-01A), and number 11 sample as the Normal group (e.g., TCGA-CV-6960-11A, TCGA-CV-7235-11A), respectively. The HNSC-related clinical information dataset and SNV (simple nucleotide variation) dataset were downloaded from the TCGA database once the sample expression matrix had been integrated, with the Data Type of the SNV dataset being Masked Somatic Mutation. The duplicated genes in GeneSymbol were deduplicated based on the mean value after the datasets had been integrated. When the gene expression values in the same sample differ greatly, the dataset would be log2-transformed. In the dataset, a gene would be excluded if its expression value is 0 in over 50% of the samples, suggesting its non-significance. Finally, samples that were present in both the expression profile and clinical information databases were extracted, and the final expression profile dataset was created based on these samples.

To search for gene sets linked to genes involved in modulating tumor cell sensitivity to T-cell mediated killing (STTK genes), details of eight high throughput screening data sets were retrieved from the TISIDB database (http://cis.hku.hk/TISIDB/index.php). Additionally, the TISDB database (http://cis.hku.hk/TISIDB/download.php) was also used to download the gene signatures of 28 tumor-infiltrating lymphocytes (TILs).

### Differentially expressed gene analysis

Differential expression analysis was conducted between the Tumor and Normal groups of HNSC using the "limma" package of the R language, where the comparison mode was Tumor vs Normal. The differentially expressed genes (DEGs) were screened based on the log2(FC) and P.adjust values in the differential analysis results. The genes with P.adjust < 0.05 and |log2FC|> 1 were selected as DEGs.

### Differentially expressed STTK (DE-STTK) genes in HNSC

The intersection of the DEG and STTK genes was identified as the DE-STTK gene and was included in the analysis that followed. First, a heat map was utilized to depict the distribution of clinical traits among samples in the tumor group as well as the expression of samples in the tumor group and normal group. Then, GO Biological process and KEGG pathway analysis of DE-STTK genes were performed using R's cluster Profiler package to observe the biological functions affected by these genes.

To obtain the effects of DE-STTK genes on other genes, the PPI (protein–protein interaction) correlation pairs of DE-STTK gene interactions were retrieved from HPRD (http://www.hprd.org/index html) and BIOGRID (http://thebiogrid.org/), and the PPI network was then constructed using the program Cytoscape (version 3.8). After the network was built, the topological characteristics of the network were examined using the Network Analyzer plug-in for Cytoscape. The Hub nodes were then filtered based on their topological characteristics. The network properties were mainly investigated using the following metrics.: Degree, Average Shortest Path Length (ASPL), Betweenness Centrality (BC), Closeness Centrality (CC), and Topological Coefficients (TC).

### Screening of Hub DE-STTK genes by combined univariate and LASSO regression analysis

To determine the essential prognostic factors in the DE-STTK genes, the expression values of DE-STTK gene were extracted in the Tumor group. Based on the clinical characteristics of OS and OS_Event, a Cox proportional hazards risk regression model (Cox-PH) was developed for each DE-STTK gene using the SURVIVAL package in R language, and the univariate analysis was performed using Cox-PH. The expression values of genes with Pvalue < 0.05 were obtained in the Tumor and Normal groups from the univariate analysis results, the genes were further filtered using LASSO (Least absolute shrinkage and selection operator) Logistic Regression. LASSO regression was utilized as a feature selection approach to identify the hub DE-STTK genes. LASSO regression performs regularization by imposing a penalty term on the regression coefficients, shrinking some coefficients toward zero while retaining predictors with non-zero coefficients in the model. For our analysis, LASSO regression was implemented using the glmnet R package with tenfold cross-validation. Tuning parameter selection was performed to identify the optimal lambda value, which controls the strength of regularization. The lambda value with minimum mean cross-validation error was selected (lambda.min). The Hub DE-STTK gene was finally acquired by using above methods. The sample risk score was established for each Hub DE-STTK gene based on univariate analysis results. Each Hub DE-STTK gene was divided into high- and low-risk groups according to the median of corresponding risk scores, and survival analysis was performed on the grouped Hub gene.

### Expression analysis of Hub DE-STTK gene in different sample groups

The expression of Hub DE-STTK gene was measured in Tumor group and Normal group, and Wilcoxon test was utilized to determine the significant correlations between Hub DE-STTK genes in the various categories. The AUC (Area Under Curve) of Hub DE-STTK genes was analyzed using ROC to assess the prediction effect of the expression value.

### Analysis of Hub DE-STTK gene associated pathway

All pathway-related genes were collected from the KEGG database (version 94.1; URL: https://www.genome.jp/kegg/pathway.html), and the Pathway containing the Hub DE-STTK gene was selected, followed by the extraction of the Pathway's non-Hub DE-STTK gene. The pathway-centric relation pairs were associated to obtain Hub DE-STK gene-Pathway relation pairs. Cytoscape (version 3.8) was used to integrate the Hub DE-STTK gene-pathway correlation pairs with the non-Hub DE-STTK gene-pathway correlation pairs, yielding the pathway-gene composite network.

### Multivariate analysis to predict the effect of Hub DE-STTK gene on HNSC survival

A hub DE-STTK genes prognostic model was developed using multivariate Cox regression analysis. The expression values of the multiple hub DE-STTK genes were extracted from the HNSCC samples in the TCGA dataset. Overall survival data including survival time and event (death) were also obtained for each patient. Cox proportional hazards (Cox-PH) regression analysis was performed using overall survival as the dependent variable and expression levels of the multiple hub genes as independent variables. This allowed estimation of a prognostic index for each patient based on a linear combination of the hub gene expressions weighted by their respective Cox regression coefficients. Expression of the multiple hub DE-STTK genes were included as covariates in the Cox model to generate a prognostic index (PI) for each patient calculated as:

$${\text{PI}}\, = \,\beta {1}*{\text{Gene1}}, + \,\beta {2}*{\text{Gene2}}, + \, \ldots \, + \,\beta {9}*{\text{Gene9}}.$$

where β1 to β9 are the Cox regression coefficients for each gene. This PI was used to compute a risk score (Risk Score = exp (PI)). Patients were stratified into high and low risk groups based on the median risk score.

To assess the predictive accuracy of the Cox-PH model, the calibration curve was generated for the time frames of 3 years, 5 years, and 10 years. The calibration procedure was performed using the calibration method implemented in the "rms" package in the R Project. The C-index value of the model was calculated as C-index = 1-C. The model was assessed using the predict method in the rms package to determine the scores of the samples, and the model scores were then processed using the rcorrcens method of the Hmisc package in the R project to determine the C-values. Finally, the ROC of the model over 3 years, 5 years, and 10 years was calculated using the timeROC package in the R project, and the ROC curve was plotted with time dependence to confirm the model effect.

Following the assessment of the model's robustness, the samples were stratified into high- and low-risk groups based on the median value of their calculated risk scores.. Then survival analysis in 3 years, 5 years, 10 years, and all samples was performed based on the sample risk score, to estimate the difference in survival between the high- and the low-risk group in different time periods.

### Hub DE-STTK in various risk groups: difference analysis

Samples from the tumor group were split into high- and low-risk categories using multivariate analysis. The Hub DE-STTK gene expression in various risk groups was then visualized using a beeswarm plot. In order to investigate the significant correlation between the two groups, a Wilcoxon test was employed to compare the data derived from distinct risk groups.

### Differences in clinical characteristics among groups with different risk

The nomogram method in the R project rms package was used to construct a nomogram to illustrate the effects of various pathological features and risk scores on survival by integrating pathological features and multivariate analysis results. Box plots were employed to examine differences between high- and low-risk groups across clinical characteristic groups and evaluate the link between risk and survival.

### Tumor mutation burden (TMB)-based analysis for the impact of Hub DE-STTK gene on HNSC

Tumor mutational burden (TMB) is the total number of somatic mutations in a tumor sample that have substitutions and insertions/deletions per Mb of exon coding regions on the genome. TMB was calculated as the total number of somatic mutations (including non-synonymous point mutations, insertions, deletions in the coding region of exons) divided by the total exon length, in mutations/Mb [14]. An increased number of mutated genes detected in the tumor tissue can result in a higher production of abnormal proteins. Consequently, this abnormal protein production may exert a more substantial influence on the overall physiological functioning of the body. Meanwhile, these abnormal proteins are more readily recognized by the immune system, thus activating the body's anti-cancer immune response and increasing the effectiveness of tumor immunotherapy.

To determine the TMB scores of the samples in the HNSC, TMB calculations were performed on the SNV dataset. The median of the sample TMB scores was computed for all samples, and the samples were then divided into High- and Low- score TMB groups based on the median. Following sample grouping, survival analysis was conducted on the samples based on OS and OS_Event to assess the correlation between TMB and survival over 3, 5, and 10 years. Finally, the expression values of Hub DE-STTK gene in HNSC samples were extracted, and the correlation between Hub gene and TMB was viewed using Pearson correlation coefficient in combination with the results of sample TMB scores.

### Correlation analysis of Hub DE-STTK gene and immune cells

The 28 immune cell gene signatures were obtained from the TISDB database [15, 16]. The specific immune cell types included: CD8 T cells, regulatory T cells, natural killer cells, macrophages, dendritic cells, etc. Gene signatures ranged from 25 to 500 genes per immune cell type, curated from literature studies profiling immune cell infiltrates. To quantify infiltration abundances, we utilized the single sample Gene Set Enrichment Analysis (ssGSEA) algorithm implemented in the GSVA R package (Version 3.17; URL: https://bioconductor.org/packages/release/bioc/html/GSVA.html). ssGSEA calculates an enrichment score representing the degree to which a gene set is overrepresented in a sample. ssGSEA was run using default parameters, with the immune cell gene signatures as input gene sets and the RNA-seq expression matrix of HNSCC samples. Significantly enriched immune cells were identified by hierarchical clustering of ssGSEA scores and visualizing using a heatmap. The Pearson correlation coefficient was then used to predict the correlation between immune cells with high abundance. High abundance immune cells were divided into two groups based on multivariate analysis, and the differences between the two groups were examined. Correlation and difference analysis was used to determine the correlation between the Hub DE-STTK gene and immune cells in HNSC.

## Results

### Identification of STTK gene aberrantly expressed in HNSC (DE-STTK)

**Fig. 2:**
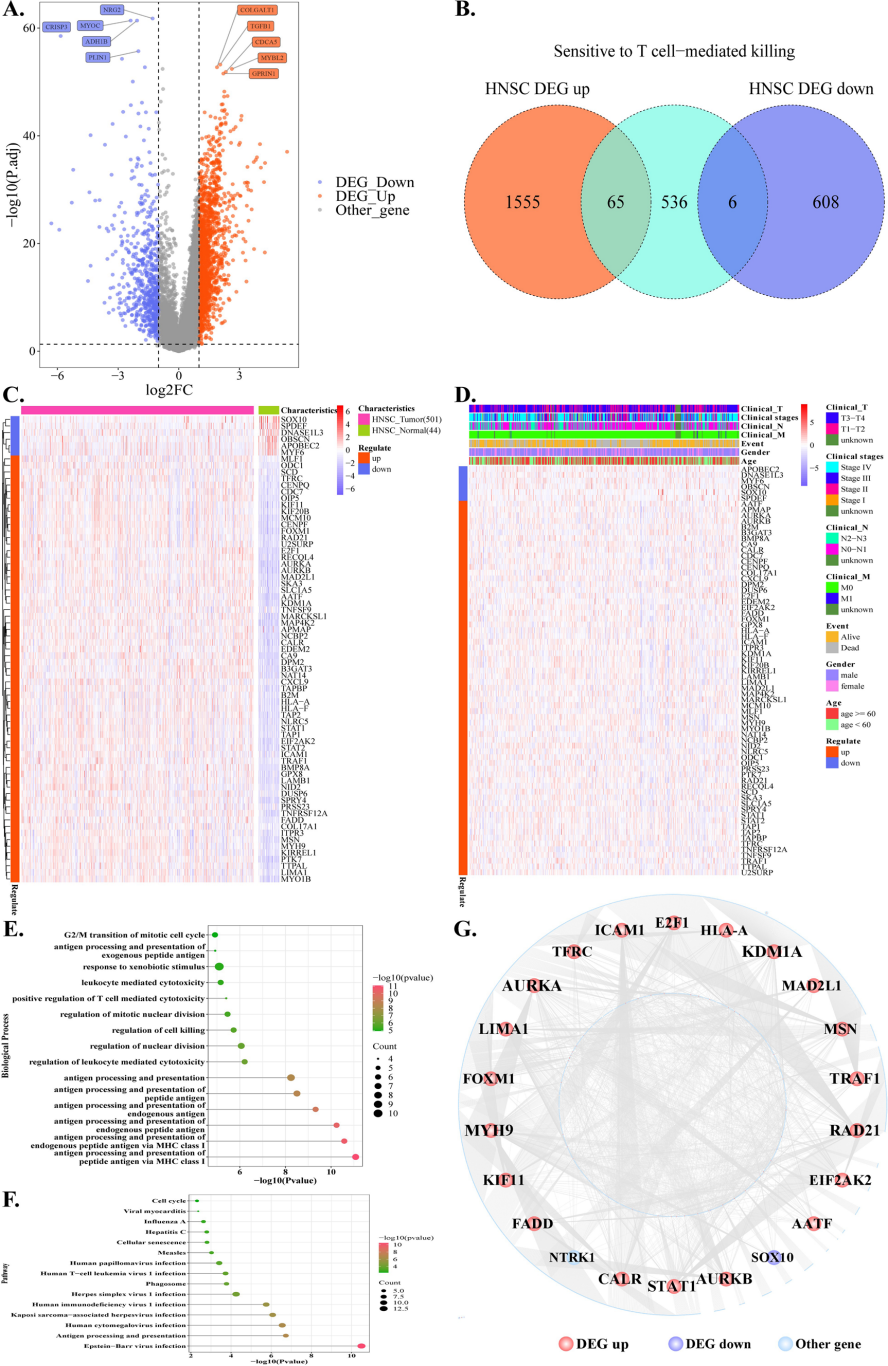
Identification of 71 DE-STTK genes and their functions. **A** Volcano plot of HNSC DEGs distribution. The Top5 genes with the most significant P.adjust values among the up-regulated and down-regulated genes are identified. **B** Venn diagram of DEG and STTK gene; **C** heat map of DE-STTK gene expression in HNSC case and control; **D** heat map of DE-STTK gene expression in various HNSC clinic features; **E** The gene enrichment analysis of 71 DE-STTK genes for biological processes; **F** Gene enrichment analysis of 71 DE-STTK genes for signaling pathway; **G** PPI network of 71 DE-STTK genes. The nodes with lower degrees have been hidden due to the large number of nodes, leaving only the Top 20 with higher degrees visible

Genes that met the criteria of having a P.adjust value of less than 0.05 and an absolute log2FC value greater than 1 were considered as DEGs. Log2FC values greater than 1 indicated up-regulation, while log2FC values less than -1 indicated down-regulation. According to the results presented in Fig. 2A a total of 2234 genes were found to be differentially expressed (DEGs). Out of these DEGs, 1620 genes were up-regulated, while the remaining 614 genes were down-regulated. The analysis of the intersection between the 2234 DEGs and the 607 STTK genes resulted in the identification of 71 DE-STTK genes. Among these, 65 genes were found to be up-regulated, while 6 genes were down-regulated. (Fig. 2B). The levels of gene expression for the 71 DE-STTK genes were collected from both the Tumor and Normal groups. Heat maps were then generated using the pheatmap package in the R language to create a visual representation of the variations in gene expression between the Tumor and Normal groups (Fig. 2C). Remarkably, the expression levels of these genes exhibited significant differences between the Tumor and Normal groups. The clinical characteristics of 71 DE-STTK genes in the Tumor group were also extracted, and the distribution of clinical characteristic expression was depicted using a heat map (Fig. 2D).

Analysis of GO Biological process and KEGG pathway for these 71 DE-STTK genes were conducted using R's clusterProfiler package, and the functionality with Pvalue < 0.05 were considered significant. After arranging all the significant pathways in ascending order based on their P values, the top 15 pathways were selected and presented in Fig. 2E, F. The analysis of DE-STTK genes revealed their prominent involvement in various biological processes, including modulation of cytotoxicity mediated by leukocytes, presentation and processing of antigens, modulation of cellular killing, and positive modulation of T cell-mediated cytotoxic responses. (Fig. 2E). Gene enrichment analysis of 71 DE-STTK genes for signaling pathway revealed that the DE-STTK genes are involved in the regulation of antigen processing and presentation, human T-cell leukemia virus 1 infection, cellular senescence pathways and other signaling pathways (Fig. 2F).

The HPRD and BIOGRID databases were combined to generate interaction gene pairs of DE-STTK genes, with 68 DE-STTK genes showing interactive correlations with other genes. The PPI network for the DE-STTK gene-interacting protein genes was developed using Cytoscape software (Fig. 2G). In total, there are 3712 nodes and 4002 edges in the DE-STTK gene PPI network. Then, topological property analysis was conducted to obtain information on the Degree parameters of each node. The topological analysis findings yielded 68 gene results, some of which were presented in Table 1. The findings indicated that MYH9, KDM1A, and AURKA are the top three hub genes in the network.

**Table 1:** Results of topological characteristics analysis for DE-STTK genes-involved PPI network

Symbol	Regulate	Degree	ASPL	BC	CC	TC
MYH9	up	311	2.734248	0.15713	0.365731	0.008039
KDM1A	up	304	2.772795	0.194186	0.360647	0.00639
AURKA	up	267	2.742402	0.153698	0.364644	0.007693
RAD21	up	251	2.901038	0.133143	0.344704	0.026371
TRAF1	up	205	2.952557	0.121228	0.338689	0.012282
LIMA1	up	182	2.843958	0.061273	0.351623	0.011898
STAT1	up	180	2.825426	0.106347	0.353929	0.009286
AURKB	up	155	2.832098	0.073164	0.353095	0.011374
ICAM1	up	143	2.978132	0.067612	0.335781	0.01288
MSN	up	127	2.800964	0.072839	0.35702	0.010155
CALR	up	124	2.907339	0.064987	0.343957	0.015195
KIF11	up	119	2.90215	0.070168	0.344572	0.010573
E2F1	up	117	2.82209	0.066303	0.354347	0.010977
EIF2AK2	up	101	2.938102	0.04678	0.340356	0.016738
HLA-A	up	76	3.023351	0.038346	0.330759	0.02203
FADD	up	72	3.031134	0.033768	0.32991	0.016815
TFRC	up	68	3.149741	0.029497	0.317486	0.054739
FOXM1	up	67	3.163084	0.027976	0.316147	0.044295
MAD2L1	up	67	3.168643	0.03177	0.315592	0.03125
AATF	up	64	3.173832	0.028306	0.315076	0.043304

### The identification of screening hub DE-STTK genes by using Univariate analysis and LASSO regression analysis

**Fig. 3:**
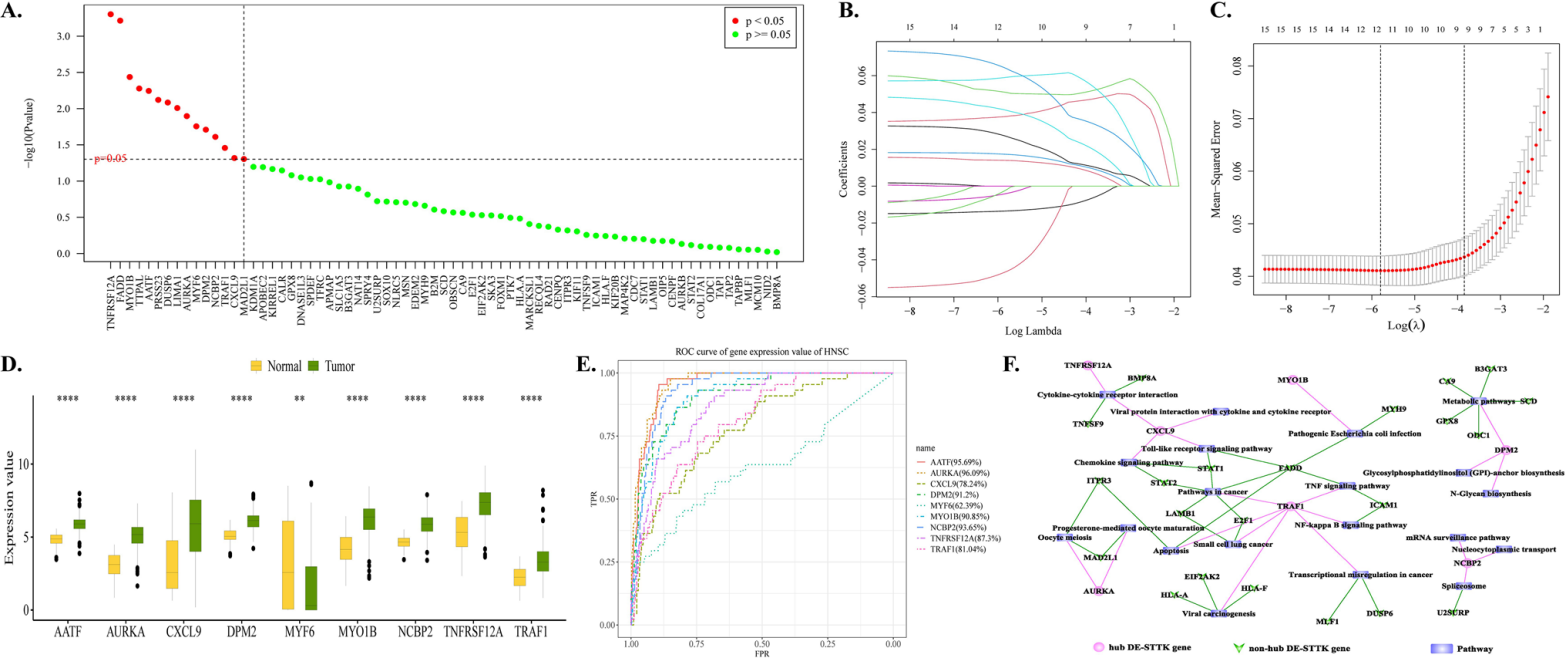
Results of Hub DE-STTK gene screening based on LASSO regression analysis. **A** The outcomes of a univariate analysis. **B** The graph depicting the LASSO analysis findings, genes are depicted as lines. As the p-value of a gene approaches 0, the corresponding horizontal coordinate (Log Lambda) value increases, indicating its greater importance. **C** The cross-validation results of the model. Dotted vertical lines were plotted at the lambda value that corresponds to the minimum mean square error (lambda.min)., and one standard error away from lambda.min (lambda.1se). The number that corresponds to the dotted line is the outcome of gene quantity screening, lambda.1se was selected as the key gene screening criterion. **D** Differential expression of Hub DE-STTK gene in Tumor and Normal group. A lower p-value in a test result indicates a more significant sample difference, and this is visually represented by an increased number of "*" on the graph. NS (not significant): p > 0.05, *: p <  = 0.05, **: p <  = 0.01, ***: p <  = 0.00, ****: p <  = 0.0001. **E** ROC prediction analysis of the hub DE-STTK gene. **F** The pathway-STTK gene network. There are 49 nodes and 55 edges in the network, with 6 hub DE-STTK genes, 20 Pathway, and 23 non-hub DE-STTK genes

Univariate analysis of the DE-STTK gene using Cox-PH resulted in 15 significant genes (Fig. 3A). The 15 DE-STTK genes were screened using LASSO (Fig. 3BC), the lambda.1se dataset in the LASSO results were extracted, and finally 9 hub DE-STTK gene were obtained (MYF6, AATF, AURKA, CXCL9, DPM2, MYO1B, NCBP2, TNFRSF12A, TRAF1). Levels of 9 hub DE-STTK gene expression were determined in Tumor and Normal samples, and the variability of the genes was analyzed between these two groups of various sample types. The findings indicated that MYF6 exhibited higher expression levels in the normal group compared to the tumor group, whereas the remaining genes exhibited elevated expression levels in the tumor group in relation to the normal group (Fig. 3D). Finally, ROC analysis was utilized to assess the predictive value of the 9 hub DE-STTK gene expression levels. From the results, all hub DE-STTK genes had good predictions (AUC > 78%) with the exception of MYF6 (AUC = 62.39%) (Fig. 3E).

From KEGG, the correlation between hub DE-STTK genes and Pathway was extracted, resulting in 6 hub DE-STTK genes and 20 Pathways. Other DE-STTK genes in the pathway were extracted and annotated as non-hub DE-STTK gene. Then, the correlation between pathway and STTK gene was analyzed and integrated and the Pathway-STTK network (Fig. 3F) was established using Cytoscape based on the construction of Pathway- STTK gene relation pairs. The Pathway- STTK network was constructed using Cytoscape (Fig. 3F). The results were obtained for multiple pathways involved in regulation by TRAF1, including apoptosis, NF-kappa B, as well as TNF signaling pathway.

### The prognostic values of hub DE-STTK genes

**Fig. 4:**
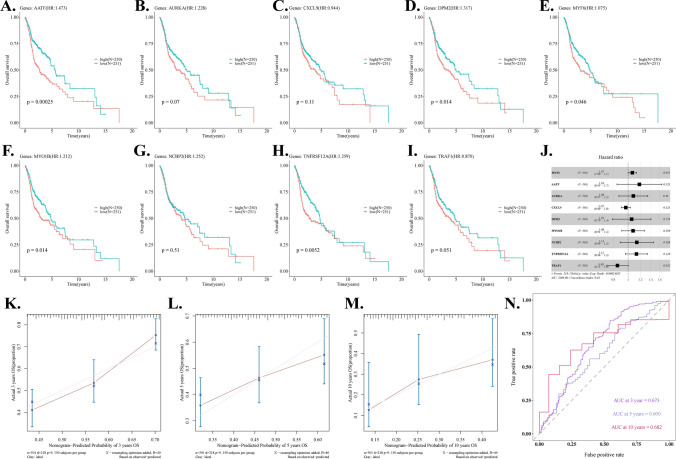
The prognostic values of hub DE-STTK genes in HNSC. Expression patterns of 9 genes in relation to overall survival outcomes in HNSC patients, including **A** AATF, **B** AURKA, **C** CXCL9, **D** DPM2, **E** MYF6, **F** MYO1B, **G** NCBP2, **H** TNFRSF12A, **I** TRAF1, respectively; **J** forest plot of Multivariate Cox regression analysis for hub DE-STTK genes; **K** Calibration curve plots of hub DE-STTK genes for 3 years; **L** Calibration curve plots of hub DE-STTK genes for 5 years; **M** Calibration curve plots for 10 years; **N** ROC analysis of hub DE-STTK genes at 3, 5 and 10 years

Based on the calculation result of the median risk score, the identified hub DE-STTK genes involved in differential signaling transduction and transcriptional regulation were stratified into high- and low-risk groups. KM curves were utilized to analyze the association between the 9 hub DE-STTK genes and survival (Fig. 4A–I). High expression of five genes (AATF (p = 0.00025), DPM2 (p = 0.014), MYF6 (p = 0.046), MYO1B (p = 0.014), and TNFRSF12A (p = 0.0052)) indicated the worse overall survival outcomes in HNSC patients; however, the respective p-values associated with the remaining 4 hub genes (AURKA, CXCL9, NCBP2, TRAF1) did not indicate statistical significance in terms of their prognostic significance in HNSC. (AURKA: p = 0.07, CXCL9: p = 0.11, NCBP2: p = 0.51, TRAF1: p = 0.051).

The levels of gene expression for the nine hub DE-STTK genes were obtained from the Tumor group. Subsequently, a Cox-PH model was constructed to perform multivariate analysis, according to the clinical characteristics OS and OS_event. The outcomes of the multivariate analysis were then summarized using forest plots, as depicted in Fig. 4J. To confirm the the predictive ability of the multivariate Cox-PH model, the "calibrate" method in the R language "rms" package was utilized to plot the calibration graph depicting the performance of the Cox-PH model at different time periods. (3 years, 5 years, and 10 years) (Fig. 4K–M). The results revealed that the model predicted better for 3 and 10 years. The model's C-index value was calculated to be 0.618. The ROC analysis approach provided a better prediction over 3 and 10 years (Fig. 4N).

### The association between risk stratification (high vs low) and survival outcomes over the course of time.

**Fig. 5:**
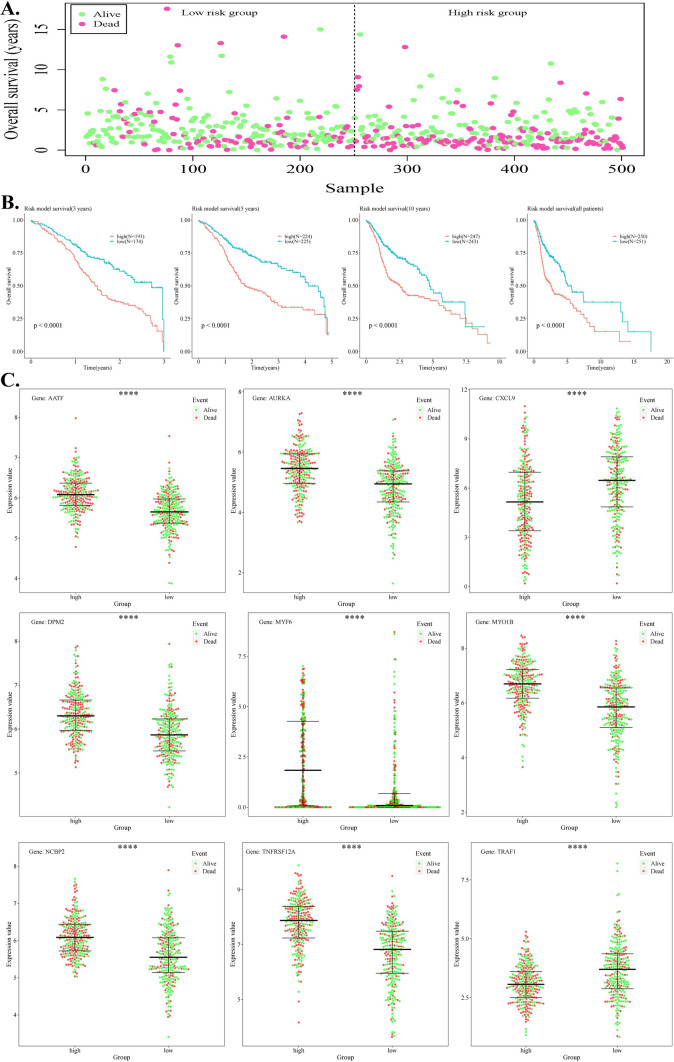
High-risk and low-risk groups exhibit different survival patterns over time. **A** Demonstration of survival time for samples in various risk groups; **B** Survival curves for various risk groups at 3, 5, 10, and all years; **C** Expression levels of hub DE-STTK genes in different risk groups

Following the assessment of the model's robustness, samples were stratified as either high- or low-risk according to their median values of their risk scores. Subsequently, the survival analysis was conducted to explore the survival outcomes of the samples in different risk groups (Fig. 5A). The findings demonstrated a greater proportion of samples classified as "dead" in the high-risk category in comparison to the low-risk group. Survival analysis was conducted independently for all samples and risk scores over 3, 5, and 10 years to evaluate if there were variations in survival rates between the high- and low-risk groups throughout the various time periods. The outcomes revealed a significant divergence in survival rates between the high- and low-risk groups as time progressed, with the high-risk group exhibiting a noticeably lower survival rate. (Fig. 5B). Based on multivariate analysis of high- and low-risk data, a swarm plot was utilized to demonstrate the expression of 9 hub DE-STTK genes in groups of samples with varying risks (Fig. 5C). Compared to the low-risk group, the results indicated a larger proportion of "Dead" samples in the high-risk group, along with reduced expressions of CXCL9 and TRAF1 genes and heightened expressions of seven other genes in the high-risk group.

### Variability in clinical characteristics across risk groups

**Fig. 6:**
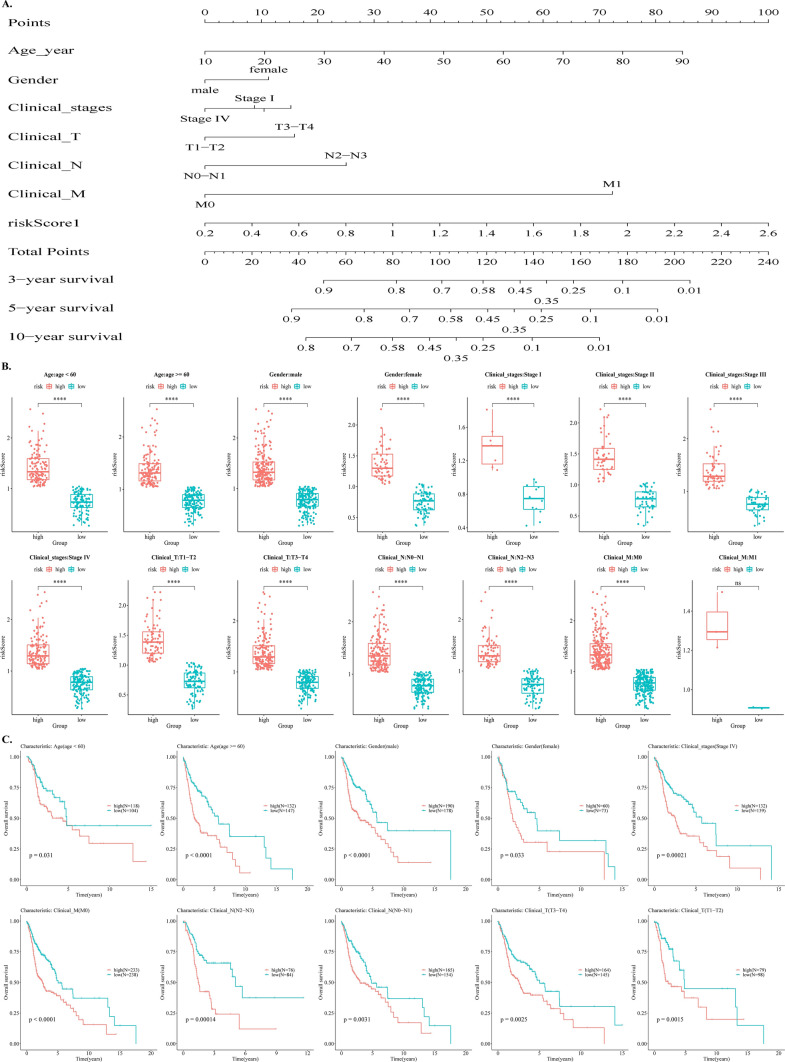
The assessment of overall survival based on the clinical characteristics. **A** The nomogram plot of clinical characteristics and risk scores. **B** The variability in different clinical characteristics of samples in various risk groups. **C** Survival correlation between high- and low- risk samples in distinct clinical characteristic groups

Integrating the clinical characteristics and conducting multivariate analysis on the 501 samples led to the 467 samples after deleting Unknown samples from Age, Gender, Clinical_M, Clinical_N, Clinical_stages, and Clinical_T. A nomogram plot was then constructed using 476 samples to illustrate the impact of distinct clinical characteristics and risk scores on survival outcomes. A patient's clinical characteristics, for example, were 60 years old (point = 53), female (point = 12), Stage 1 (point = 10), T3 (point = 15), N2 (point = 25), and M1 (point = 72). A comprehensive evaluation yielded a total point of 187 for the patient. Based on total points, this patient's survival probability at 3 years is approximately 0.1, approximately 0.05 at 5 years, and no survival status at 10 years. Valid samples were extracted from 501 samples in the groups with various clinical characteristics. The valid samples in the various clinical characteristics categories are displayed in Table 1. For instance, in Clinical_T, there are 177 valid samples in the T1-T2 group, 309 good samples in the T3-T4 group, and 15 invalid samples. Box plots were created for each valid sample group to show the variations in clinical characteristics among the groups at high- and low-risk (Fig. 6B). The results demonstrated significant variations in various clinical characteristics in the context of high-risk versus low-risk groups (Fig. 6B). The association between different risk groups within various clinical characteristics and survival was then reviewed, and the groups of characteristics with Pvalue < 0.05 are shown in Fig. 6C. The results revealed significant variations in terms of survival between groups categorized as high-risk and low-risk, within groups with varying clinical characteristics (Fig. 6C).

### Identification of hub DE-STTK genes and Tumor mutation burden (TMB) of HNSC

**Fig. 7:**
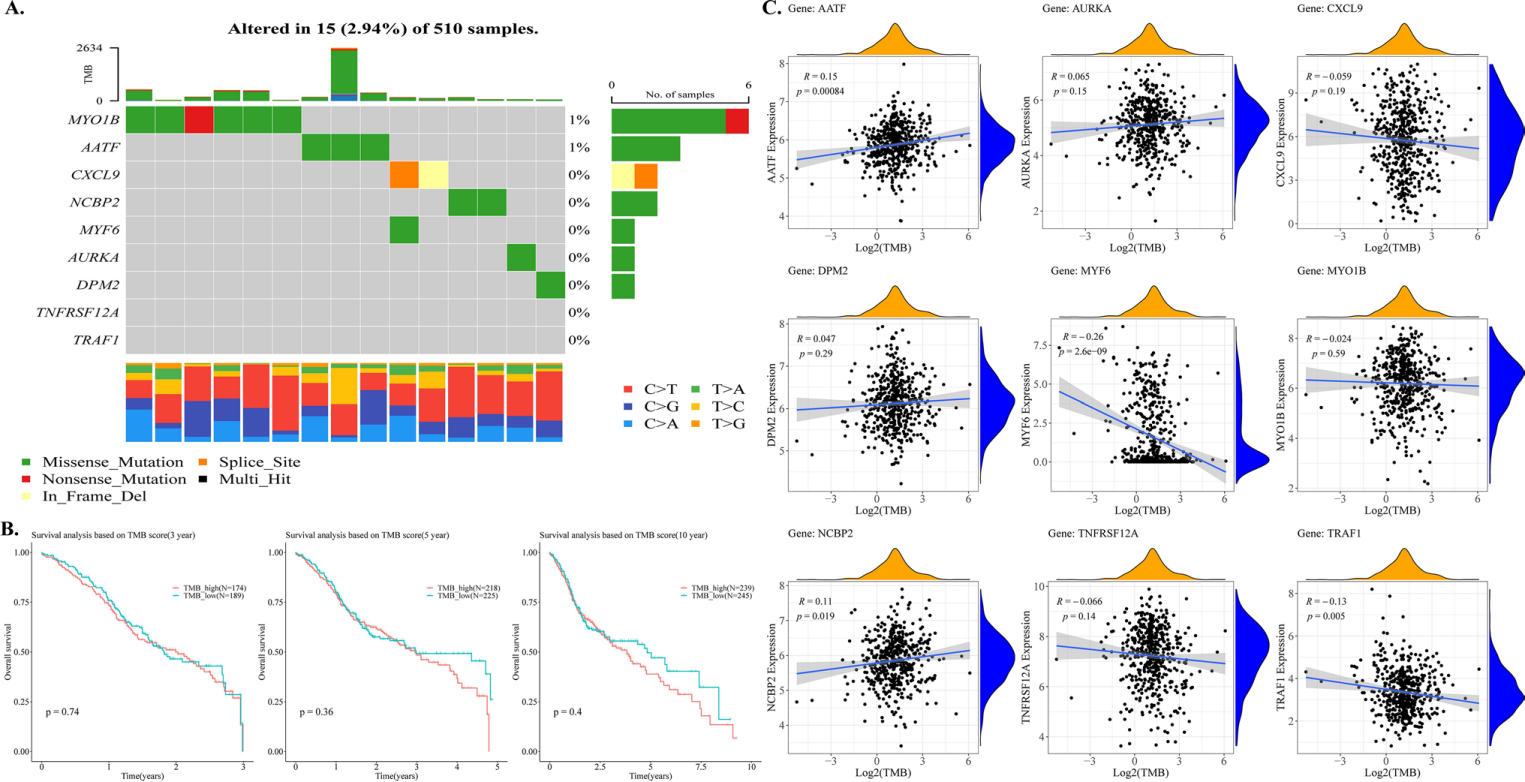
The correlation between TMB and survival. **A** Mutation waterfall plot of the hub DE-STTK genes; The horizontal and vertical axes indicate samples and genes, respectively, and the right-side scale represents the proportion of samples having gene mutations to total samples. The grey squares indicate unmutated samples, whereas the other colours represent mutated samples, with the type of mutation indicated in the legend. The bar at the top of the image represents statistics for all mutated genes and the type of mutation in each sample, while the bar on the right indicates the count of samples with mutations in the current gene and the bar at the bottom shows base alterations in each sample. **B** The correlation between high- and low- TMB groups and survival. **C** Correlations between the DE-STTK gene and the TMB

The SNV information of the hub DE-STTK genes was extracted, and a waterfall plot was created for display using the R language maftools package (Fig. 7A). The overall mutation rate of these genes in HNSC was 2.94%, according to the findings. MYO1B gets mutated frequently in HNSC, and the majority of its mutation types are Missense mutations. The TMB score was then calculated using the SNV dataset, and the categorization of all samples into high- and low- TMB groups was accomplished by utilizing the median TMB scores as the pivotal threshold. TMB results (510 samples), OS, OS_Event, and 9 Hub gene expression matrices (501 samples) were integrated to obtain 495 valid samples. KM curves were utilized to investigate the link between TMB and survival at 3, 5, and 10 years (Fig. 7B). The results revealed no statistically significant discrepancies in survival outcomes in the high versus low TMB group comparison. Subsequently, the Pearson correlation coefficient was employed to examine the link between the Hub DE-STTK genes and TMB status. It was discovered that MYF6, AATF, NCBP2, TRAF1, and TMB were significantly related with TMB (Fig. 7C), and no significant correlation was observed between TMB and other hub genes.

### Enrichment analysis of immune cells in HNSCs

**Fig. 8:**
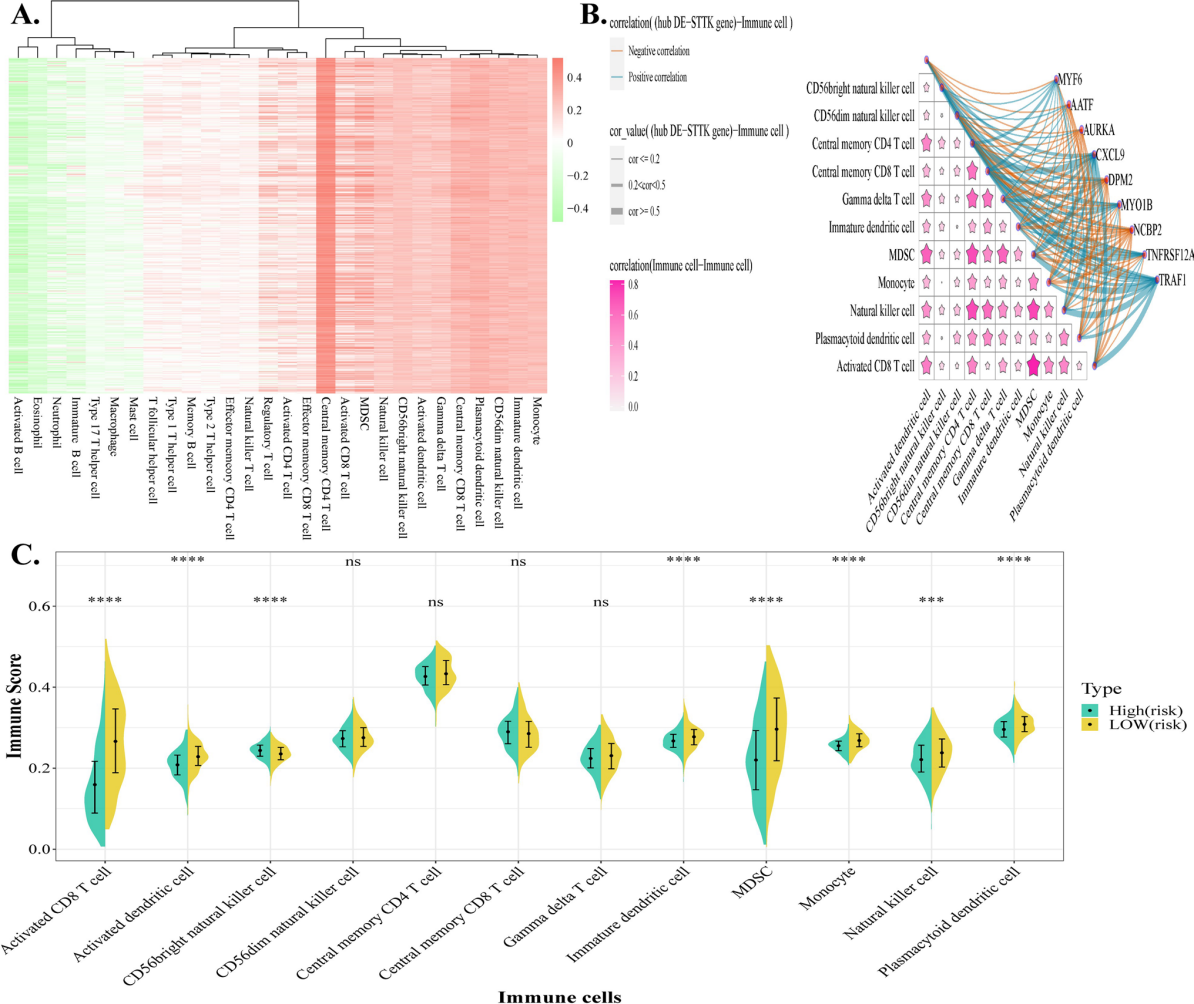
Enrichment analysis of immune cells. **A** Significant enrichment of immune cells in HNSC; **B** correlation of hub DE-STTK gene and immune cells with high abundance; **C** The expression levels of immune cells with high abundance in various risk groups

From the TISDB database, a total of 28 distinct immune cell species and a collection of 782 genes associated with immune cells was obtained. The enriched abundance of HNSC-associated immune cells was then determined using ssGSEA quantitative analysis of 501 tumour samples. The abundance scores of immune cells in the samples from the Tumor group were visualized using a heat map, and the enriched Immune cells with high abundance were derived using hierarchical clustering. The results showed a significant enrichment of the following immune cells: Activated dendritic cell, CD56bright natural killer cell, CD56dim natural killer cell, Central memory CD4 T cell, Central memory CD8 T cell, Gamma delta T cell, Immature dendritic cell, MDSC, Monocyte, Natural killer cell, Plasmacytoid dendritic cell, and Activated CD8 T cell (Fig. 8A). Subsequently, the Pearson correlation coefficient was utilized to assess the relationship between immune cells with high abundance, as well as the association between immune cells with high abundance and the hub gene.. The results demonstrated that the majority of correlations between 9 hub DE-STTK genes and immune cells were positive. Among them, high correlations were discovered between MDSC and activated CD8 T cells, MDSC and natural killer cells, MDSC and central memory CD4 T cells, natural killer cells and central memory CD4 T cells (Fig. 8B). NCBP2, TRAF1, and CXCL9 were also identified with a high correlation to immune cells. By employing multivariate analysis, the study investigated the differential analysis of high abundance immune cells among different subgroups of samples. The findings revealed that 8 immune cells with high abundance exhibited significant differences across various risk groups (Fig. 8C).

## Discussion

The primary findings of this study identified 9 hub DE-STTK (MYF6, AATF, AURKA, CXCL9, DPM2, MYO1B, NCBP2, TNFRSF12A, and TRAF1) and their implicated signaling pathways (Toll-like receptor signaling, cytokine-cytokine receptor interaction, TNF signaling, NF-kappa B signaling, spliceosome, mRNA surveillance pathway, nucleocytoplasmic transport, GPI-anchor biosynthesis, and N-Glycan biosynthesis).

CXCL9 (Chemokine (C-X-C Motif) Ligand 9) generated by myeloid cells within the TMB was found to regulate T cells migration via attracting activated T cells expressing the CXCR3 receptor [17]. In tumor-bearing mice, CAR T cells modified with CXCL9 displayed enhanced infiltration of immune cells by attracting a greater number of T cells to the tumor site. [18]. The CXCL9 involved signaling axis (CXCL9, -10, -11/CXCR3) was found to suppress tumor progression by regulating immune cell migration, differentiation, and activation [19] and further promoting anti-tumor immune response. The expression levels of CXCL9 were found to be significantly higher in TCGA_HNSC tumor samples in comparison to the healthy control samples, as depicted in Fig. 3D. The results of CXCL9 expression pattern in serum showed the consistent tendency: The serum concentration of CXCL9 displayed a notable elevation among patients with nasopharyngeal carcinoma in contrast to those in the control group of healthy individuals [20]. Figure 3F shows that CXCL9 was implicated in the Toll-like receptor signaling pathway. A significant increase in Toll-like receptors (TLR)-4,6,9,10 expression was observed in T-regulatory (T-reg) cells specimens derived from peripheral blood mononuclear cells (PBMCs) obtained from patients with HNSC compared with healthy donors [21]. The activation of T-reg cells by engaging TLR4 in patients with HNSC has the potential to bolster the inhibitory capabilities of T-reg cells., thereby potentially contributing to tumor-induced immune suppression [21].

Tumor necrosis factor receptor superfamily member 12A (TNFRSF12A), is the smallest receptor member within the TNF superfamily. Unlike other members, TNFRSF12A does not possess a cytoplasmic death domain. Additionally, its upregulation has been observed in cases of HNSC [22]. The suppression of TNFRSF12A gene expression resulted in the inhibition of HNSC cell line (CAL-27) cell proliferation and migration [23]. TNFRSF12A silencing was found to promote the stimulation and expansion of T cells and further inhibit the cell viability of gastric cancer cell lines [24]. A noticeable correlation was found between increased expression levels of the immunosuppressive gene TNFRSF12A and a notable decline in the survival rate among individuals with HNSC [25]. Immunotherapy with anti-TNFRSF12A antibodies might be effective in treating HNSC patients by attracting effector immune cells [26]. Figure 3F shows that TNFRSF12A is involved in the cytokine-cytokine receptor interaction pathway. Cytokines such as IL-12 and IFN-gamma was found to promote the transition of T cells into effector T cells through differentiation., which possess the ability to identify and eliminate tumor cells [27]. Conversely, other cytokines such as TGF-beta and IL-10 were discovered to inhibit T cell activation and contribute to immune suppression [28]. The signaling pathway involved in the interaction between cytokines and cytokine receptors (cytokine-cytokine receptor pathway) is implicated in regulating T cell-mediated immune checkpoint pathways, such as the PD-1/PD-L1 and CTLA-4 pathways [29]. In the context of HNSC, implementation of cytokine-based therapy with IL-2, IL-7, and IL-15 has demonstrated the ability to augment T cell activation, expansion, and anti-tumor effector function in patients [30].

The overexpression of another hub gene TRAF1 (TNF Receptor-Associated Factor 1) was found to inhibit apoptotic cell death of CD8 ( +) T lymphocytes triggered by antigens in tumor transgenic mice [31]. TRAF1 was found to negatively regulate TNFR2-mediated proliferative signals in T cells. As depicted in Fig. 3F, the involvement of TRAF1 was observed in the NF-κB signaling pathway. NF-κB has been recognized as a critical controller of T-cell homeostasis, including processes such as T-cell activation, expansion, viability, functionality, and expression of distinct cytokines [32]. The initiation of NF-κB signaling cascade was found to promote upregulation of PDL1 levels in NPC (nasopharyngeal carcinoma) cells to support immune evasion [33]. The promoted expression of PDL1 can enhance the suppressive function on the T lymphocytes activation [34]. In addition, TRAF1 was also involved in the viral carcinogenesis pathway. HPV-positive HNSC displayed a robust Th1 response, in contrast to HPV-negative HNSC, as indicated by the enhanced infiltration of diverse immune cell subtypes and upregulated expression of T-cell exhaustion markers. HPV-positive HNSC exhibited a T-cell-inflamed phenotype and consequently demonstrated a positive outcome in terms of prognosis in comparison to HPV-negative HNSC [35].

The findings depicted in Fig. 3F highlight the role of the hub gene DPM2 (Dolichyl-Phosphate Mannosyltransferase Subunit 2, Regulatory) in metabolic pathways, particularly Glycosylphosphatidylinositol (GPI)-anchor biosynthesis and N-Glycan biosynthesis. Glycolysis was shown to be highly upregulated in HNSC [36]. The glycolytic metabolism is a contributing factor of disease progression and found to decrease sensitivity to radiation or chemotherapy in HNSC [36]. The glycolysis synthesis as a key metabolic pathway is essential for T cell immunometabolism. Modulation of the metabolic programme of T cells might be an encouraging avenue to improve to enhance antitumor immunity and enhance the effectiveness of adoptive T cell-based immunotherapies [37]. The glycolytic metabolism in T cells typically facilitates the trigger of effector T cell response, consequently fostering the polarization towards an anti-tumor Th1 phenotype [38]. The inhibition of glycolytic metabolism has been identified as a strategy that facilitates the development of long-lived memory CD8 + T cells, consequently augmenting anti-tumor immune responses and bolstering anti-tumor immunity [39]. In addition, both GPI-anchor biosynthesis and N-glycan biosynthesis have been demonstrated to participate in the tumor immunity. GPI-anchored proteins on tumor cells can bind to CD48 on T cells, which in turn leads to T-cell co-stimulation and activation [40]. Additionally, GPI anchors on tumor cells can also modulate the function of Tregs, which play a critical role in suppressing anti-tumor immunological responses [40]. The process of N-glycan biosynthesis has been identified to participate in the modulation of T-cell receptor (TCR) signaling and consequently enhance the subsequent activation of T-cells. N-glycans impede the development of Th1 or Th17 cells from T helper cells, and promote the differentiation of regulatory Tregs; all of which are able to suppress anti-tumor immunity [41].

Another hub gene NCBP2 is found to be involved in spliceosome, mRNA surveillance pathway, and nucleocytoplasmic transport. Spliceosome mutations have been suggested to affect the immune response against head and neck cancer by altering the splicing pattern of genes involved in T cell immunity and function [42, 43]. Specifically, spliceosome mutations might lead to aberrant splicing of key molecular players (e.g., CD44, CTLA-4, and CD300) in T cell activation and function, resulting in impaired T cell activation and function [44, 45]. The study conducted by Giraldo found that mutations in genes involved in RNA surveillance were associated with decreased T cell activity and poor clinical outcomes in individuals afflicted with clear cell renal cell carcinoma [46]; however, there is still no research showing the mRNA surveillance pathway in tumor immunity of head and neck cancer. Impairments in nucleocytoplasmic transport can lead to aberrant gene expression and the development of T cell exhaustion, which is characterized by reduced T cell function and impaired anti-tumor immunity [47].

The hub genes AATF, AURKA, and MYO1B are implicated in various mechanisms contributing to the pathogenesis and evolution of HNSC. The research by Fu et al. demonstrated that the upregulation of AATF in HNSC is linked to tumor cell apoptosis and chemoresistance, potentially mediated through the regulation of STAT3/survivin signaling [48]. AURKA, as a mitotic kinase involved in cell cycle regulation, exhibits high expression in HNSC and is implicated in the process of epithelial-mesenchymal transition, drug resistance, oncogenic signaling, and stem cell characteristics, thereby promoting the development of invasive disease [49]. MYO1B, a myosin motor protein, is closely involved in cellular movement and migration. Overexpression of this Class I myosin promotes the development of a more migratory phenotype [50]. Previous research has shown that its high expression in HNSC facilitates tumor cell migration [51, 52]. These hub genes play a crucial role in the development and progression of HNSC by modulating multiple processes, including tumor cell proliferation, migration, invasion, and stemness.

The tumor microenvironment (TME) is essential for immunotherapy resistance in HNSC. Head and neck tumors tend to have fewer tumor infiltrating lymphocytes (TILs) compared to other tumor types, indicating an immunosuppressive TME. Factors within the TME such as hypoxia, altered metabolism, and the release of immunosuppressive cytokines can hinder the function of T cells. and promote resistance to immunotherapy. Additionally, increased presence of suppressive immune cells in the TME of head and neck tumors is associated with poorer responses to immunotherapy. Strategies to overcome these TME-mediated resistance mechanisms, such as combining immunotherapy with therapies that modulate the TME, are being explored to improve outcomes in HNSC [8, 9]. In this study, elevated expression of the STTK genes is linked to immune cell infiltration, such as effector T cells and dendritic cells, in tumor tissues. This implies that the STTK gene may increase the sensitivity of tumor cells to T cell-mediated killing by promoting immune cell infiltration. Several genes involved in pathways associated with activation of T cells, trafficking, and effector functions were identified. For example, CXCL9 is a chemokine that attracts effector T cells expressing CXCR3 into the tumor microenvironment [19]. TNFRSF12A and TRAF1 are involved in TNF and NF-kB signaling pathways, which are crucial for T cell proliferation and survival [53]. However, in contrast to the increased immune cell infiltration caused by the STTK gene, the TME can diminish the sensitivity of tumor cells to T cell-mediated killing by inducing immune suppressive cell infiltration and promoting T cell dysfunction [54, 55]. Therefore, this manuscript provides evidence for the impact of the STTK genes on modulating the sensitivity of tumor cells through immune cell regulation. However, the immune suppression induced by the tumor microenvironment presents a significant counteracting force against the actions of the STTK gene, and together they determine the sensitivity of tumor cells to T cell killing. A comprehensive understanding of this dynamic balance would contribute to the development of precise immunotherapy approaches.

The limitation of the current study needs to be emphasized. This study is purely based on data analysis and computational models and lacks direct experimental validation, which limited the reliability of the findings identified in the current research. This study is limited in scope as it relies on a restricted sample size from the TCGA database for analysis. The generalizability and applicability of the results should be interpreted with caution since not all of the results identified in the current study can be validated by referring to the previous literature. To enhance the robustness of the findings, future research should consider adopting a multi-center design that encompasses a diverse population comprising different ethnicities and geographical regions. Furthermore, experimental validation is warranted to substantiate the reliability of the computational results. Integrating multi-omics datasets may offer a more comprehensive elucidation of the underlying mechanisms involved. It is important to note that the current findings primarily establish correlations, and further mechanistic investigations are necessary to establish the direct impact of the identified genes and pathways. By considering these recommendations in future studies, a more precise comprehension of the pathogenesis of HNSC and the functional roles of the identified key genes can be achieved. Additionally, it is worthwhile to mention the potential future implications that identifying the key STTK genes will bring for combating HNSC. On the one side, the identification of such genes can offer insight into personalized immunotherapeutic approaches that target specific genes or pathways in HNSC. On the other side, the key STTK genes identified in the current research can pave the way for developing genetic biomarkers which can be used for predicting patients more likely to benefit from immunotherapy; and for developing more effective strategies for overcoming immune evasion and enhancing anti-tumor immunity.

## Conclusion

In summary, we identified 9 hub STTK genes (i.e., MYF6, AATF, AURKA, CXCL9, DPM2, MYO1B, NCBP2, TNFRSF12A, and TRAF1) that played vital functions in the tumor immunity of HNSC. The signaling pathways enriched by these hub STTK genes are verified by previous literature evidence to be implicated in tumor immunity by modulating the immune response of T cells in HNSC. The identification of crucial STTK genes might serve as a useful guidance and aid for clinicians in the personalized immunotherapy approaches for individuals with HNSC.

## Data Availability

The data analyzed during the current study are available in TCGA database with the accession numbers TCGA-HNSC. The original contributions presented in the study are included in the article; further inquiries can be directed to the two equally corresponding authors.
